# Purification and Partial Characterization of Agglutinin Lectin from Heamolymph of German Cockroach, *Blattella germanica*

**DOI:** 10.18502/jad.v14i2.3732

**Published:** 2020-06-30

**Authors:** Zohreh Nabavi, Mozhgan Baniardalani, Hamidreza Basseri

**Affiliations:** Department of Medical Entomology and Vector Control, School of Public Health, Tehran University of Medical Sciences, Tehran, Iran

**Keywords:** German cockroach, Heamolymph, Lectin

## Abstract

**Background:**

Lectin molecules have crucial biological role in insects’ immune system. The aim of present study was to find the agglutinin activities in haemolymph of German cockroach, *Belatella germanica* with appropriate screening and purification.

**Methods:**

The heamolymph of cockroach was collected and agglutinin test performed against different animal and human red blood cells (RBC). Then sugar inhibition assay was carried out to find carbohydrate specific binding lectin. The proteins of haemolymph was purified using ion-exchange chromatography (HPLC) and each fraction was tested for agglutinin activity. Finally the molecular weight of the agglutinin protein was determined using SDS-page.

**Results:**

The most agglutinin activity of haemolymph was found against RBC of mouse at titer 1/128ml/L dilution and sugar inhibition assay showed that fucos, N-acetyglucoseamine and galactose reduced titer of agglutinin to ½ml/L. Only one fraction of heamolymph at rotation time of 36 minute showed agglutinin activity. The molecular weight of this lectin was measured as 120Kds.

**Conclusion:**

The range of agglutinin activities against different RBC indicates that the isolated lectin is not specific for a particular carbohydrate. In addition, the isolated lectin at low concentration present in heamolymph should be an innate lactin not secreted, because we found it without any trigger immunity of the insect.

## Introduction

Insects lack specific acquired nonself recognition systems such as antibody in vertebrates, and lectins molecules have main role of non-self recognition in the innate immune response (1–4). Lectins can agglutinate foreign matter and thus become to be it more suitable for understanding. These molecules can also mediate haemocytes invasion to non-self particles, and consequently cause lysis, increase phagocytosis of foreign particles and activate the coagulation system (5, 6).

Lectins are proteins or glycoproteins that recognize specifically and bind reversibly the carbohydrate-containing molecules of foreign cells (7–10). Lectin molecules have a range of immunologic action including antitumor, antifungi and bacteria activities which may find practical applications (11, 12). Carbohydrate-binding lectins with similar properties are presence on the surfaces of many pathogens. Therefore, a wide variety of pathogens can be recognized by specific binding of lectins (13, 14). However, the interaction between lectins and carbohydrates as ligand receptors is common in living organisms (15).

It has been shown that two C-type lectins has roles in antibacterial defense as well as in the melanization response to *Plasmodium berghei* in *Anopheles gambiae* (16). In addition, lectins as immune substances have been reported in American cockroach (*Periplaneta americana*) and desert locusts (*Shistocerca gregaria*) (17–19). Multiple lectins have been purified from only two species of cockroaches, namely, the American cockroach, *P. americana*; and the West Indian leaf cockroach, *Blaberus discoidalis* (20, 21). In addition, fucose-binding lectin in the heamolymph of *P. americana* showed the maximum phenoloxidase activity against dopamine (6). According to our knowledge and survey of literatures, there are no many references exist about agglutinin activity in the heamolymph of German cockroach, *Blattella germanica*. Therefore, in the present study, we tried to identify and purify lectin molecule s from heamolymph of German cockroach. Our results provide a new insect lectin from *B. germanica*. It seems C-type lectin as receptors provide main role for penetration of cockroach allergens (22). This is the first report on characterization of hemagglutinin activity in German cockroach heamolymph. Hence, in the present study, we also attempt to isolate and purify lectin from the heamolymph of *B. germanica*. The finding of current study provides initial information about presence of lectin the heamolymph of German cockroach. These results provide preliminary information for further studies such as allergenic actives cause by German cockroach.

## Materials and Methods

### Insect rearing and bleeding

German cockroaches, *Blatella germanica*, were maintained in an insectary at 25±2 °C with humidity of 60–65%, and fed on dried bread, sugar cubic with water. Adult cockroaches (450 insects) were anaesthetized with CO_2_ and then the ventral surface of sternum of each cockroach was sterilized with 70% ethanol. The coxa membrane and ventral joint of abdomen to thorax were pierced with a sterile needle to pull out haemolymph. The haemolyphm was collected, homogenized and then centrifuged at 1800 rpm for 15 minutes. The supernatant was kept in −80 °C until used. The protein concentration of each sample was measured by Bradford assay method (23).

### Haemagglutination assay

Haemagglutination assays were performed presented by Chen et al. (24). The red blood cells (RBCs) of rabbit, rat, sheep, Guinea pig, Syrian mouse and human (A, B, AB and O groups) were used to exam haemagglutination activities of the haemolymph of *B. germanica*. All RBCs were washed three times in 10mM Phosphate Buffered Saline (PBS) containing 150mM NaCl and 10mM sodium phosphate at pH 7.4 and 380mOsm, 1mM CaCl2 at 4 °C and the concentration of the RBCs suspensions to the buffer and adjusted to final concentration of 2%.

Five microliters of the buffer was dispensed in each microtiter plate well and then two folded serial dilution of the haemolymph extract was prepared in each row of plates to give final dilution ranges of 2^−1^ to 2^−10^, prior to the addition of 5μl RBCs to each well. The plates were covered and kept at room temperature for two hours. The end points of agglutination examined under a stereomicroscope and by the flow characteristics of the erythrocyte pellets when the plate was held at an angle. All experiments were replicated three times and the controls always included PBS plus RBCs alone.

### Carbohydrate inhibition assays

Carbohydrate inhibition assays were followed as mentioned by Chen et al. (24). The sugar specificities of cockroach lectins were investigated by competitive binding using the following carbohydrates: D-(+)-glucose, D-(+)-galactose, D-(+)-mannose, L-(−)-fucose, lactose, N-acetyl-D-glucosamine, N-acetyl-D-galactosamine, fructose and arabinose (all purchased from Sigma Co.). Stock solutions of sugars were prepared in PBS at 0.2M and stored in −4 °C until use. Two folded serial dilutions of haemolymph (each of 5μl) were prepared in PBS followed by addition of 5μl of appropriate carbohydrate at the above initial concentration. The plates were incubated at room temperature for 60min and then 5μl of the mouse RBCs added to the respective wells. The controls were consisted of carbohydrate plus PBS, and plus RBCs alone. Inhibitory effects were recorded as those reducing agglutination in the wells. The end points of agglutination were examined under a stereomicroscope and by the flow characteristics of the erythrocyte pellets when the plate was held at an angle. This experiment was replicated three times.

### Lectin purification

Ion exchange-High Performance Liquid Chromatography (HPLC) (Knauer Co., Germany) was utilized to separate protein from extracted haemolymph. The samples once more were centrifuged for 15 minutes at 1800 rpm and supernatant filtered three times through a 0.45μm filter. The sample was loaded to column resin (Asahi chemical Co., Japan) with carboxymethyl as the functional group as particles, 7.5×100 ID, 150mm length, 2000 Pore size). The mobile phase was 1M of NaCl in 20mM Tris-HCl (pH= 7.5–8) which applied at a flow rate of 1ml/min at room temperature. The column was previously equilibrated with 20mM Tris-HCl buffer at volume 300μL from 0–20min and a flow rate of 1.0ml/min. The sample at 300μL volume was loaded to an ion-exchange column. The proteins were eluted with a 50min linear gradient of 90–10% NaCl/Tris-HCl at a flow rate of 1.0ml/min at room temperature. The chromatographic run was monitored at 280 nm of absorbance.

The fractions were collected manually and then the eluted fractions were concentrated using freeze-dryer device. Finally agglutinin activity of each eluted protein was assayed. The molecular weight of agglutinin protein was measured by polyacrylamide gel electrophoresis (SDS-PAGE).

### Polyacrylamide gel electrophoresis (SDS-PAGE)

The molecular weight of protein, purity and molecular mass of fractions were estimated using SDS-page. Tris base 30.3g, Glycine 144g, SDS 10g and make to 1L with dH_2_O. Twenty μL of the eluted protein were loaded on the SDS-PAGE gels (12%) and run. The gel was stained with Coomassie brilliant blue R 250/Silver. As well as standard protein marker was loaded on SDS-PAGE (mix of seven proteins, 14.4–116KDa). BioRad apparatus was used, the gels were run at 200V (constant voltage) until the bromophenol blue dye was just off. Gels were run at room temperature and 50–60 minutes.

### Ethics approval

Ethics approval was not required for this study.

## Results

### Haemagglutination assay

**Fig. 1.**
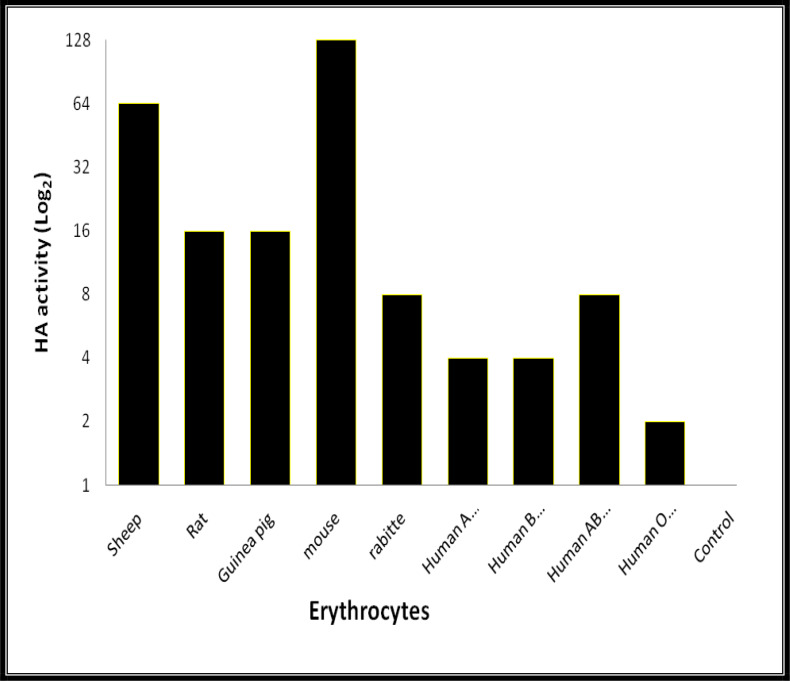
Agglutination activities of haemolymph of the German cockroach, *Blattela germanica*, against different red blood cell (RBC) at 2% concentration in phosphate buffered saline. All experiments were replicated three times and the control was Phosphate Buffered Saline (PBS) free haemolymph

The results of haemagglutination activities of whole cockroache serum against a range of erythrocytes were represented in Fig. 1. The highest activity were found against mouse erythrocytes (titer ≤128) followed by Sheep (titer ≤ 64) but these activities were relatively less against different human erythrocytes (Fig. 1). The lowest activity was observed with human O-erythrocytes (titer ≤8). Thus, Mouse erythrocytes were candidate for further inhibition assays.

### Carbohydrate inhibition assay

**Fig. 2.**
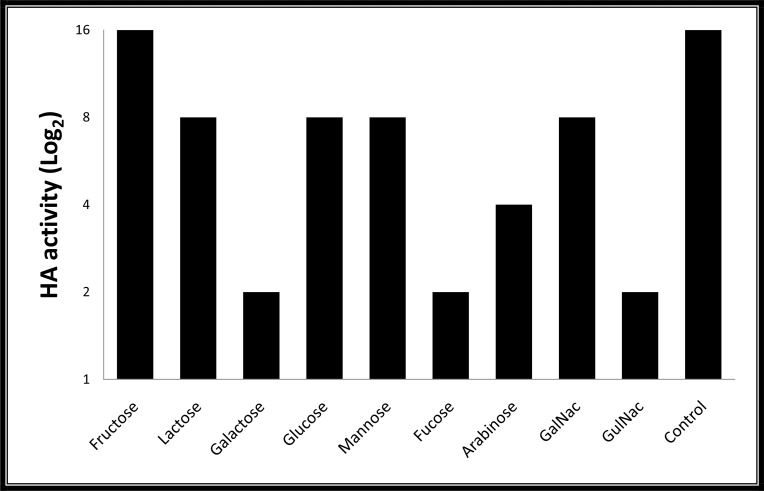
Haemagglutination inhibition assay of hemolymph lectins of the German cockroach, *Blattela germanica*. The assay was performed with mice Red Blood Cells (RBCs) (2%) in Phosphate Buffered Saline (PBS). The control was haemolymph free carbohydrate

The results of haemagglutination inhibition assay are represented in Fig. 2. The haemolymph lectin activity was reduced by all tested sugars except fructose. The inhibitory effect of Galactose, fucose and N-acetyl-D-glucosamine was more than other carbohydrates while the agglutination of hemolymph reduce to titer of ≤2. Fructose did not show any specific binding to lectins of haemolymph. Glucose, mannose, lactose and N-acetyl-D-galactosamine showed similar inhibitory effect on the lectin activity at titer of ≤8 which indicate that non-specific binding exist in the haemolymph lectins for these carbohydrates. Arabinose also blocked agglutinin activity at titer of ≤ 4 which it was moderate inhibitor.

### Ion-exchange Chromatography

**Fig. 3.**
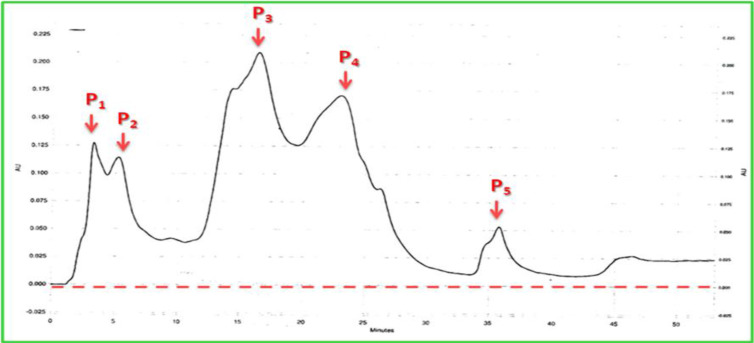
Ion exchange-High Performance Liquid Chromatography (IE-HPLC) of *Blattela germanica* hemolymph. 300μL of the diluted hemolymph was loaded into the column (7.5×100 ID, 150mm length, 2000 A° Pore size). The column was equilibrated with 300μL water from 0–20min at a flow rate of 1.0ml/min. Proteins were eluted with a 50min linear gradient of 90–10% NaCl / Tris-HCl at a flow rate of 1.0ml/min at room temperature. The chromatographic run was monitored at 280nm of absorbance. Five peaks were observed in chromatogram, Only fifth peak was determined presence of lectin in hemagglutination assay

**Fig. 4.**
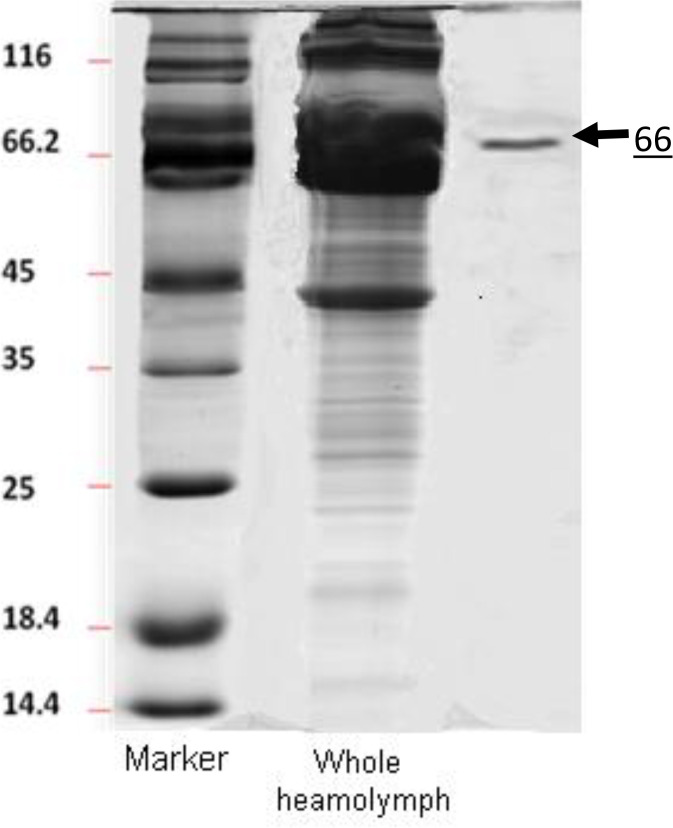
Sodium dodecyl sulphate-polyacrylamide gel electrophoresis (SDS-PAGE) of whole heamolymph proteins of *Belattella germanica* and the fractions which showed agglutinin activity. All fractions prepared using Reversed phase High Performance Liquid Chromatography (RP-HPLC) and applied for agglutinin activity assay. The molecular weight of isolated lectin was approximately 66KDa. Gel was stained with Coomassie brilliant blue R 250

Following the separation and purification of the protein molecules using ion exchange HPLC, five peaks at chromatogram were appeared. All fractions were applied for haemagglutination activities and the main activity was found by fifth peak which appeared at 36min retention time (t_R_) in chromatogram (Fig. 3). The molecular weight of the purified protein was estimated to be about 66 kDa by sodium dodecylsulfate polyacrylamide gel electrophoresis (Fig. 4).

## Discussion

In the current study, we assessed haemagglutinin activity in the heamolymph of German cockroach. The mouse and sheep erythrocytes showed high agglutinin reaction with the heamolymph lectins. Similarity, the erythrocytes of mice showed high agglutinin activity against heamolymph of discoid cockroach, *Blaberus discoidalis*. Therefore, the authors used the mice erythrocytes for study on two isolated lectins called as BDL1 and BDL2 (24). In the current study, we also used mouse erythrocytes for inhibition assays.

German cockroaches have developed effective innate immunity of protecting themselves against pathogenic microorganisms including epidermal immunity (25) or humoral immune defense (24).

Lectins are one of the pattern-recognition proteins concerned in innate immunity in insects (4). The presence of lectin activities in the heamolymph is more important for immunity of *B. germanica* against microbial pathogens. As recently showed, C-type lectin has crucial role to response to pathogen infection by the expression of antimicrobial peptides and the agglutination of bacteria immunity in red flour beetle *Tribolium castaneum* (3). Therefore, further work needs to characterize the role of lectins in immunity of the German cockroach.

We optimized ion exchange-HPLC to purify that protein which showed agglutinin activities. The multiple lectins had been purified from different species of cockroaches e.g. the American cockroach, *Periplaneta americana* (6, 19, 25–27) and the West Indian leaf cockroach, *Bl. discoidalis* (21). At least four lectins have been reported in this species, namely, BDL1, BDL2, BDL3, and GSL (24). In addition, based on carbohydrate specificity, we characterized specificity of protein binding carbohydrates to the hemolymph lectins of the German cockroaches using different carbohydrates. The lectins of German cockroach showed more affinity to glucose, N-acetyl-D-gluctosamine and fucose. Based on Makela category (28), in the current study, the lectin in *B. germanica* heamolymph could be categorized in groups II and III. These types of lectins are more involve in immunity of insects and have crucial role in phagocytosis of microorganisms (20).

In addition, the heamolymph lectin of *B. germanica* agglutinated different types of animal and human blood cells which indicated this lectin is non-blood type–specific. Generally, lectins recognized α-linked on carbohydrate surface of red blood cells, as we could not find RBC specificity, it may indicate the lectins failed to distinguish between α and β-linked of carbohydrates on the surface of the blood cells. It was also possible that the lectin have independent binding sites, which can be used for attachment to the erythrocyte surface.

In the present study, only one fraction of the heamolymph with molecular weight of 66KDa (with no subunits) showed agglutinin activities against mice RBC (Fig. 1). Generally, the molecular weights of all lectins of invertebrates vary from 26KDa to 1500KDa (29–31). This range of difference could be depended on species or methods used for purification, analysis and protocols. The maximum molecular weight of insect lectin (1500 KDa) was reported in *P. americana* (25) whereas the lowest molecular weight lectin was that of *Agrotis segetum* with 69KDa and no subunits (32) while we introduce a lectin from heamolymph of *B. germanica* with molecular weight of 66KDa. According to our results, this agglutinin activities could be calcium dependant-lectin. While the C-type lectin family consists of members that bind their ligands in a calcium-dependent manner, many other C-type lectins show the hemoagglutinin activity in a calcium-independent manner.

Conclusively, our current results provide a new insect lectin from German cockroach, *B. germanica*. This finding would be helpful in future studies on lectins concerning.
